# Deep learning reconstruction for improved image quality of ultra-high-resolution brain CT angiography: application in moyamoya disease

**DOI:** 10.1007/s11604-025-01806-5

**Published:** 2025-05-29

**Authors:** Yongping Ma, Satoshi Nakajima, Yasutaka Fushimi, Takeshi Funaki, Sayo Otani, Miyuki Takiya, Akira Matsuda, Satoshi Kozawa, Yasuhiro Fukushima, Sachi Okuchi, Akihiko Sakata, Takayuki Yamamoto, Ryo Sakamoto, Hideo Chihara, Yohei Mineharu, Yoshiki Arakawa, Yuji Nakamoto

**Affiliations:** 1 Department of Diagnostic Imaging and Nuclear Medicine, Kyoto University Graduate School of Medicine, 54 Shogoin-Kawahara-Cho, Sakyo-Ku, Kyoto, 606-8507 Japan; 2 Department of Neurosurgery, Kyoto University Graduate School of Medicine, 54 Shogoin-Kawahara-Cho, Sakyo-Ku, Kyoto, 606-8507 Japan; 3 Division of Clinical Radiology Service, Kyoto University Hospital, 54 Shogoin-Kawahara-Cho, Sakyo-Ku, Kyoto, 606-8507 Japan; 4 Department of Applied Medical Imaging, Gunma University Graduate School of Medicine, 3-39-22 Showa-Machi, Maebashi, Gunma 371-8511 Japan; 5 Department of Artificial Intelligence in Healthcare and Medicine, Kyoto University Graduate School of Medicine, 54 Shogoin-Kawahara-Cho, Sakyo-Ku, Kyoto, 606-8507 Japan

**Keywords:** Deep learning reconstruction, Ultra-high-resolution CT, Brain CT angiography, Moyamoya disease

## Abstract

**Purpose:**

To investigate vessel delineation and image quality of ultra-high-resolution (UHR) CT angiography (CTA) reconstructed using deep learning reconstruction (DLR) optimised for brain CTA (DLR-brain) in moyamoya disease (MMD), compared with DLR optimised for body CT (DLR-body) and hybrid iterative reconstruction (Hybrid-IR).

**Materials and methods:**

This retrospective study included 50 patients with suspected or diagnosed MMD who underwent UHR brain CTA. All images were reconstructed using DLR-brain, DLR-body, and Hybrid-IR. Quantitative analysis focussed on moyamoya perforator vessels in the basal ganglia and periventricular anastomosis. For these small vessels, edge sharpness, peak CT number, vessel contrast, full width at half maximum (FWHM), and image noise were measured and compared. Qualitative analysis was performed by visual assessment to compare vessel delineation and image quality.

**Results:**

DLR-brain significantly improved edge sharpness, peak CT number, vessel contrast, and FWHM, and significantly reduced image noise compared with DLR-body and Hybrid-IR (*P* < 0.05). DLR-brain significantly outperformed the other algorithms in the visual assessment (*P* < 0.001).

**Conclusion:**

DLR-brain provided superior visualisation of small intracranial vessels compared with DLR-body and Hybrid-IR in UHR brain CTA.

## Introduction

Moyamoya disease (MMD) is a cerebrovascular steno-occlusive condition characterised by progressive stenosis of the terminal portion of the internal carotid artery and the formation of an abnormal network of fragile perforators at the base of the brain [1]. Moyamoya perforator vessels in the basal ganglia and periventricular anastomosis are small but significant. Periventricular anastomosis is a unique phenomenon occurring in MMD and is classified into lenticulostriate, thalamic, and choroidal types. Among these, the choroidal type is a strong predictor of intracranial bleeding [2–4].

Brain CT angiography (CTA) has emerged as a promising alternative to digital subtraction angiography and MR angiography. It is useful for identifying vessel location and geometry, and its short acquisition time makes it favourable for emergency situations associated with MMD, including cerebral haemorrhage, aneurysm, and stroke [5, 6]. Accurate delineation of small vessels is essential but remains a challenge in brain CTA. Small intracranial vessels are more susceptible than large vessels to the noise and limited spatial resolution of CT systems. Ultra-high-resolution (UHR) CT offers a solution to these challenges. Its smaller detector elements and larger reconstruction matrix than conventional multidetector-row CT enable production of images with higher spatial resolution [7, 8]. A previous study has reported that UHR CTA in MMD provided vessel visualisation comparable to that of digital subtraction angiography [9].

Despite its superior spatial resolution, one drawback of UHR CT is increased image noise, which cannot be mitigated conventionally without an increase in radiation dose [10]. To reduce image noise, iterative reconstruction (IR) techniques such as Hybrid-IR and model-based IR (MBIR) have been utilised in clinical CT systems [11]. MBIR is more effective than Hybrid-IR for the delineation of small vessels [12]; however, long reconstruction times have limited its clinical application. Deep learning reconstruction (DLR) is a newly developed algorithm for CT reconstruction [13], and a deep convolutional neural network (CNN) kernel trained with MBIR images offers markedly reduced calculation time [14]. In previous studies involving abdominal, coronary, pulmonary, and lower extremity CTA images, DLR has been reported to improve vessel delineation [15–21]. However, few studies have applied DLR to brain CTA [22, 23].

We hypothesised that UHR CTA, when combined with DLR, would be suitable for MMD studies that typically require evaluation of small vessels. In a previous study, comparison of DLR optimised for body CT (DLR-body) with Hybrid-IR in UHR brain CTA for MMD failed to demonstrate superiority in vessel delineation [9]. The present study aimed to apply a new DLR algorithm optimised for brain CTA (DLR-brain) to UHR brain CTA for MMD, and to investigate image quality and vessel delineation of DLR-brain in comparison with DLR-body and Hybrid-IR.

## Materials and methods

This retrospective study was approved by the local institutional review board, which waived the need for written informed consent.

### Scanning parameters and image reconstruction

All CT examinations, including the phantom experiment, were performed using UHR CT (Aquilion Precision; Canon Medical Systems, Otawara, Japan). CTA protocols for patients employed super-high-resolution mode with a 1024 × 1024 reconstruction matrix.

For paediatric patients (age < 15 years), the scanning parameters were as follows: tube voltage, 100 kV; automatic exposure control for tube current; pitch factor, 0.569; rotation speed, 0.75 s; focus size, 0.6 × 0.6 mm (the second smallest of the six focal sizes); detector configuration, 0.25 mm × 160 rows/1792 channels. A 24-gauge catheter was positioned in an antecubital vein, and 25 mL of contrast medium at 370 mgI/mL (Iopromide; FUJIFILM Toyama Chemical, Tokyo, Japan) was injected at 1 mL/s using the bolus tracking method.

For adult patients, tube voltage was 120 kV, tube current was 260 or 310 mA, and 50 mL of Iopromide was injected at 4 mL/s using a 20-gauge catheter.

CT images were reconstructed from the raw data with the following parameters: slice thickness, 0.25 mm; slice interval, 0.25 mm; and display field of view, 210 mm. The reconstruction kernels used were FC44 with Hybrid-IR (Adaptive Iterative Dose Reduction 3D, enhanced standard setting; Canon Medical Systems), DLR-body (Advanced Intelligent Clear-IQ Engine [AiCE], BodySharp standard setting; Canon Medical Systems), and DLR-brain (AiCE, BrainCTA standard setting; Canon Medical Systems). AiCE trains deep CNNs with low-dose Hybrid-IR images as input and MBIR images as ground truth. Reconstruction times for a representative case with 641 CT images were 37.4 s for Hybrid-IR, 45.9 s for DLR-body, and 50.4 s for DLR-brain.

The images were reformatted as maximum intensity projection (MIP) with a 10-mm slab thickness in the coronal plane. Post-contrast images were transferred to a workstation (RapideyeCore; Canon Medical Systems) to create whole-brain MIP images.

### Phantom experiment

A phantom experiment was conducted to evaluate and compare the noise characteristics and spatial resolution of the three reconstruction algorithms. A cylindrical TOS phantom (Canon Medical Systems) with a diameter of 180 mm was used in this study. The same slice in the TOS phantom was scanned 10 consecutive times using the following parameters: tube voltage, 120 kV; tube current, 310 mA; pitch factor, 0.569; rotation speed, 0.75 s; slice thickness, 0.25 mm; slice interval, 0.25 mm; field of view, 210 mm; detector configuration, 0.25 mm × 160 rows/1792 channels; image matrix, 1024 × 1024. The volume computed tomography dose index (CTDIvol) was 53.8 mGy.

The noise power spectrum (NPS) corresponding to the spatial frequency was calculated and used to represent the noise characteristics. The NPS helps to characterise how noise is distributed across different spatial frequencies, which affects the visual appearance of noise in the image. A square (256 × 256) region of interest (ROI) was placed at the centre of the phantom images. The NPS was calculated from 0.01 to 2.34 cycles/mm as the average of 10 images. A task-based modulation transfer function (TTF) was then calculated using the circular edge method to evaluate spatial resolution. The TTF describes how well the imaging system can reproduce the contrast of various spatial frequencies present in the object being imaged. The analysis was performed using integrated CT measurement software (CTmeasure version 0.99 d; Japanese Society of CT Technology) [24].

### Patients

Brain CTA was performed on 100 consecutive patients with suspected or diagnosed MMD between January 2018 and July 2020. We enrolled 50 patients and excluded 50 patients due to (1) different contrast media (*n* = 6), (2) different injection volumes or rates (*n* = 30), (3) different radiation doses (*n* = 8), and (4) unavailable datasets (*n* = 6).

### Quantitative analysis

**Fig. 1:**
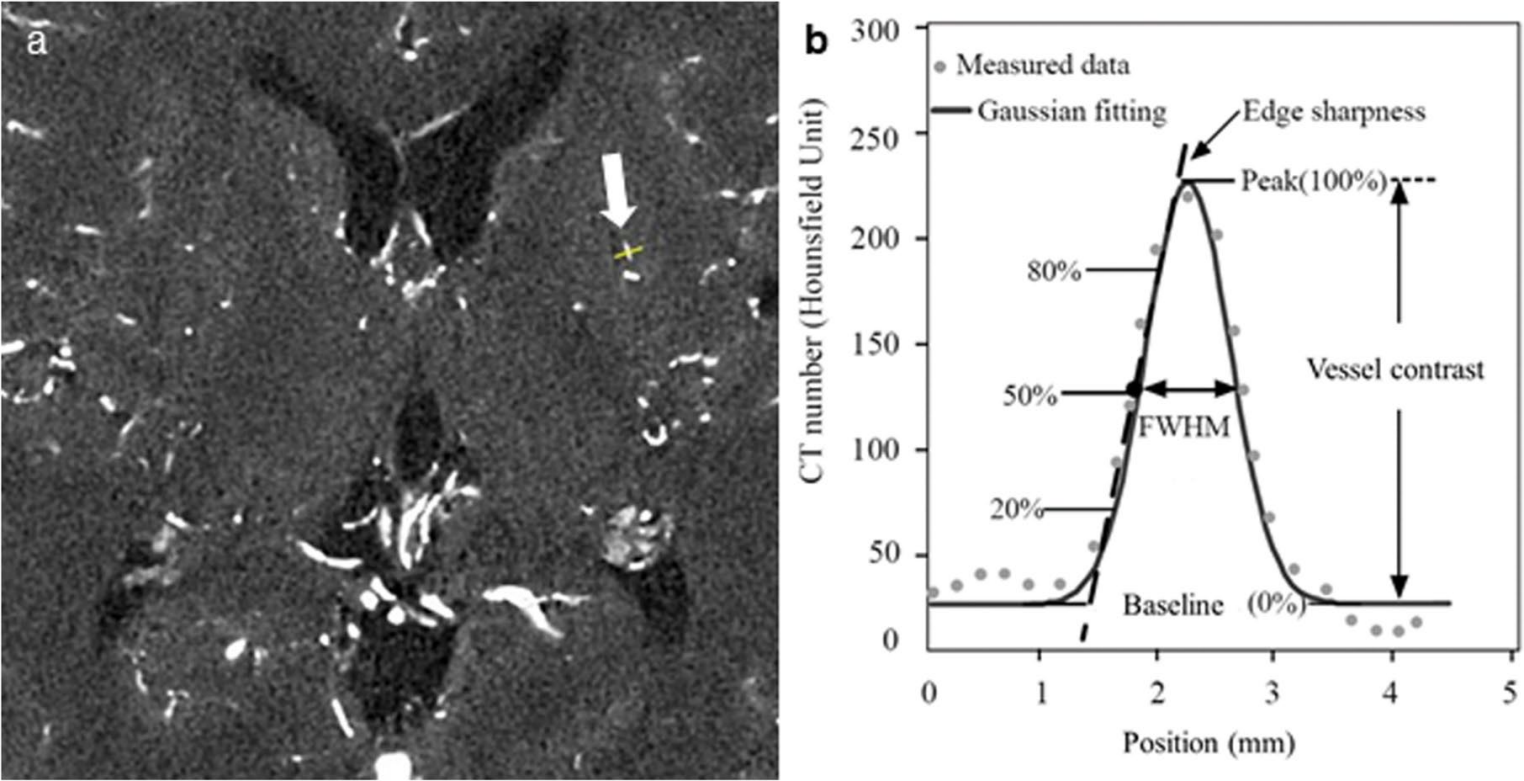
Measurements of edge sharpness, peak CT number, vessel contrast, and FWHM. **a** A linear region of interest is positioned perpendicular to the target vessel on the source image. The arrow indicates a moyamoya perforator vessel. The locations of regions of interest varied among the patients.** b** Measurements were taken on the Gaussian-fitted line profile. Edge sharpness was defined as the slope of a line passing through 20% and 80% of the peak CT number. Vessel contrast was defined as the difference between the peak (100%) and baseline (0%) CT numbers. *FWHM* full width at half maximum

We evaluated moyamoya perforator vessels in the basal ganglia and periventricular anastomosis using a calculation method based on profile curves. A linear ROI was set on a cross-sectional image using ImageJ version 1.54 d (National Institutes of Health) [25] (Fig. 1a). The linear ROI was drawn using ImageJ’s straight-line tool. ROI location varied among the patients. We selected the thinner moyamoya vessels wherever possible. ROIs were placed by a board-certified radiologist (Y.M., with 10 years of experience in neuroradiology) and approved by another board-certified radiologist (S.N., with 18 years of experience in neuroradiology). Measurements were taken on the Gaussian-fitted line profile. Edge sharpness, peak CT number, vessel contrast, and full width at half maximum (FWHM) were then measured from the profile curve according to the definitions shown in Fig. 1b. The baseline of the profile curve reflects the CT numbers of the surrounding brain parenchyma and serves as a reference for comparison with the CT numbers of the target vessel. Since the development and diameter of moyamoya perforator vessels are not uniform between the right and left sides, these were analysed separately.

Image noise was defined as the standard deviation (SD) of attenuation in a ROI (size, 20 mm^2^) placed in the pons and white matter of the frontal lobes. To avoid including moyamoya vessels, ROIs were not placed in the basal ganglia or thalamus.

### Qualitative analysis

Two board-certified radiologists (S.O. and M.T., with 13 and 7 years of experience in neuroradiology, respectively) who were blinded to the reconstruction algorithms and patient demographics reviewed all CTA slab MIP images. These images were managed using the workstation and displayed on liquid–crystal display monitors (RadiForce GS521; EIZO, Hakusan, Japan) in random order. Both raters evaluated the following aspects of the target vessels: vessel delineation, noise magnitude, noise texture, and overall quality.

Vessel delineation was scored as grade 1 (blurry), grade 2 (slightly blurry), grade 3 (moderate), grade 4 (good), or grade 5 (sharpest). Noise magnitude and texture were scored as grade 1 (excessive noise, impaired evaluation), grade 2 (severe noise, reduced diagnostic quality), grade 3 (moderate noise, acceptable for routine diagnosis), grade 4 (minimal noise, good diagnostic quality), or grade 5 (barely perceived noise, excellent diagnostic quality). Overall quality was scored as grade 1 (poor), grade 2 (suboptimal), grade 3 (acceptable), grade 4 (good), or grade 5 (excellent). Disagreements in the visual scores were resolved by consensus.

### Statistical analysis

Statistical analysis was performed using MedCalc version 22.021 (MedCalc Software). The Friedman test was used to compare the values of all parameters (edge sharpness, peak CT number, vessel contrast, FWHM, SD, vessel delineation, noise magnitude, noise texture, and overall quality). For subsequent multiple comparisons, Wilcoxon signed-rank tests with Bonferroni correction were conducted. A *P*-value < 0.05 was considered statistically significant. Inter-rater agreement for subjective scores was evaluated using Cohen’s kappa coefficient.

## Results

### Phantom experiments

**Fig. 2:**
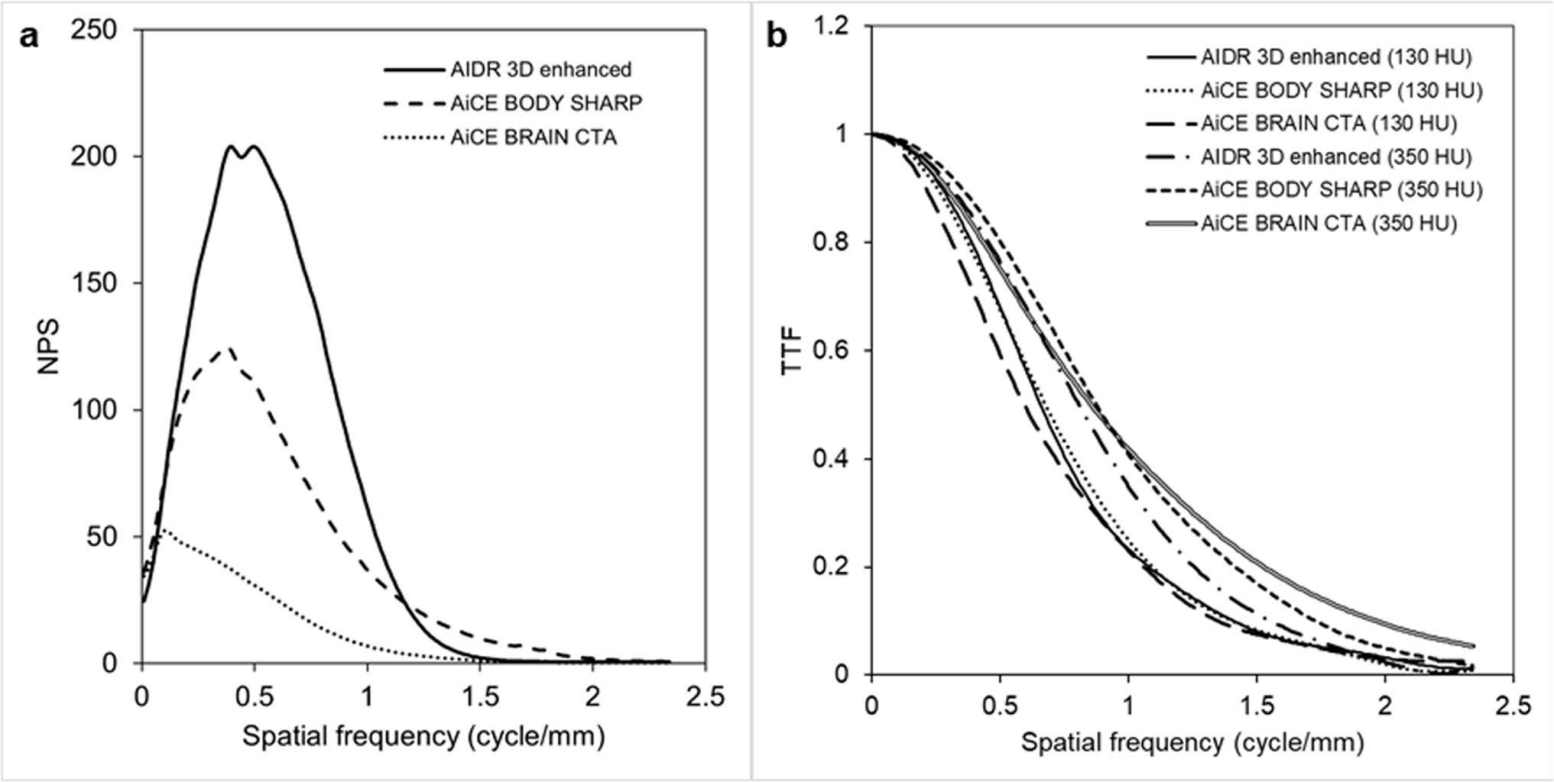
Results of phantom experiments.** a** NPS curves of DLR-brain (AiCE BrainCTA standard setting), DLR-body (AiCE BodySharp standard setting), and Hybrid-IR (AIDR 3D enhanced standard setting). **b** TTF curves of DLR-brain, DLR-body, and Hybrid-IR. *AiCE* Advanced Intelligent Clear-IQ Engine, *AIDR* Adaptive Iterative Dose Reduction, *DLR* deep learning reconstruction, *IR* iterative reconstruction, *NPS* noise power spectrum, *TTF* task-based modulation transfer function

The NPS curves are shown in Fig. 2a. DLR-brain markedly reduced image noise across the spatial frequency domain.

The TTF curves are shown in Fig. 2b. The TTF results obtained using the circular section within the phantom that exhibited contrast of 130 HU showed no significant difference among the three algorithms. However, for the circular section with contrast of 350 HU, the TTF of DLR-brain was the highest when the spatial frequency exceeded 1 cycle/mm, which corresponds visually to objects smaller than 0.5 mm.

### Patient characteristics

This study included 50 patients (20 males and 30 females: 16 paediatric patients, mean age 7.0 ± 2.1 years; 34 adult patients, mean age 43.8 ± 11.3 years).

### Quantitative analysis

Table 1 lists the edge sharpness, peak CT number, vessel contrast, and FWHM values of the target vessels. For all vessels, edge sharpness was significantly higher for DLR-brain than for DLR-body and Hybrid-IR (*P* < 0.001). For all vessels except those of the left basal ganglia, edge sharpness was significantly higher with Hybrid-IR than with DLR-body (*P* < 0.01).

**Table 1:** Results of quantitative analysis

Parameter	Hybrid-IR	DLR-body	DLR-brain
Edge sharpness			
Right basal ganglia	206.1 ± 72.6*^#^	179.9 ± 65.7*	262.8 ± 131.9
Left basal ganglia	241.4 ± 111.6*	227.2 ± 124.9*	308.7 ± 133.5
Periventricular anastomosis	266.3 ± 116.1*^#^	240.1 ± 121.3*	329.7 ± 139.7
Peak CT number			
Right basal ganglia	163.0 ± 32.2*^#^	150.9 ± 33.0*	175.3 ± 45.9
Left basal ganglia	185.4 ± 52.0*^#^	176.9 ± 54.4*	202.2 ± 54.4
Periventricular anastomosis	213.4 ± 70.4*^#^	198.0 ± 69.9*	230.2 ± 72.7
Vessel contrast			
Right basal ganglia	122.5 ± 34.4*^#^	109.6 ± 35.3*	136.0 ± 47.9
Left basal ganglia	141.0 ± 52.9*^#^	133.6 ± 55.3*	154.5 ± 54.3
Periventricular anastomosis	177.7 ± 72.3*^#^	163.5 ± 71.1*	195.9 ± 74.6
FWHM			
Right basal ganglia	0.80 ± 0.24*	0.81 ± 0.23*	0.70 ± 0.15
Left basal ganglia	0.80 ± 0.22*	0.80 ± 0.19*	0.69 ± 0.14
Periventricular anastomosis	0.88 ± 0.20*	0.90 ± 0.20*	0.78 ± 0.16
SD			
Pons	24.3 ± 3.8*^#^	18.7 ± 1.9*	9.5 ± 1.6
White matter	22.8 ± 4.2*^#^	17.2 ± 1.3*	8.6 ± 1.3

Continuous variables are expressed as the mean ± SD

*DLR* deep learning reconstruction, *FWHM* full width at half maximum, *IR* iterative reconstruction, *SD* standard deviation

^*^Indicates a significant difference from DLR-brain (*P* < 0.05)

^#^Indicates a significant difference from DLR-body (*P* < 0.05)

For all vessels, peak CT number and vessel contrast values were significantly higher for DLR-brain than for DLR-body and Hybrid-IR (*P* < 0.05). For all vessels, peak CT number and vessel contrast values were significantly higher for Hybrid-IR than for DLR-body (*P* < 0.01).

For all vessels, FWHM was significantly greater for DLR-brain than for DLR-body and Hybrid-IR (*P* < 0.001). There was no significant difference in FWHM between Hybrid-IR and DLR-body.

Image noise, represented by SD, was measured in the pons and white matter (Table 1). Image noise was significantly lower for DLR-brain than for DLR-body and Hybrid-IR (*P* < 0.001). Image noise was significantly lower for DLR-body than for Hybrid-IR (*P* < 0.001).

### Qualitative analysis

**Fig. 3:**
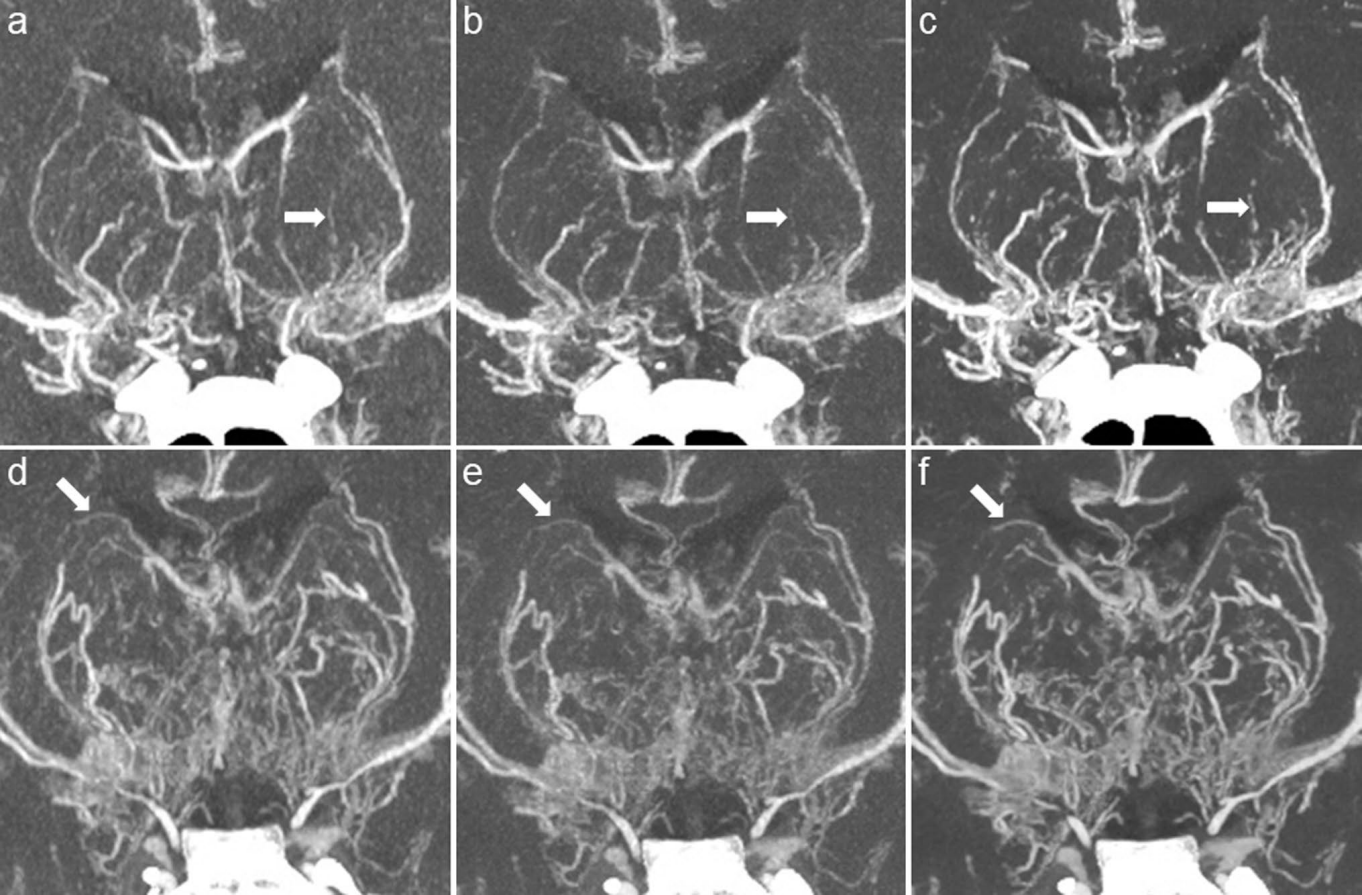
**a–c** A 41-year-old woman with moyamoya disease. **d–f** A 3-year-old boy with moyamoya disease (post-bilateral STA-MCA bypass surgery). Coronal slab MIP images reconstructed using Hybrid-IR (**a, d**), DLR-body (**b, e**)**,** and DLR-brain (**c, f**). All images show moyamoya perforator vessels in the basal ganglia. Some of these vessels appear obscure or blurred in the Hybrid-IR and DLR-body images, whereas they are distinct in the DLR-brain images (partly indicated by arrows). *DLR* deep learning reconstruction, *IR* iterative reconstruction, *MCA* middle cerebral artery, *MIP* maximum intensity projection, *STA* superficial temporal artery

**Fig. 4:**
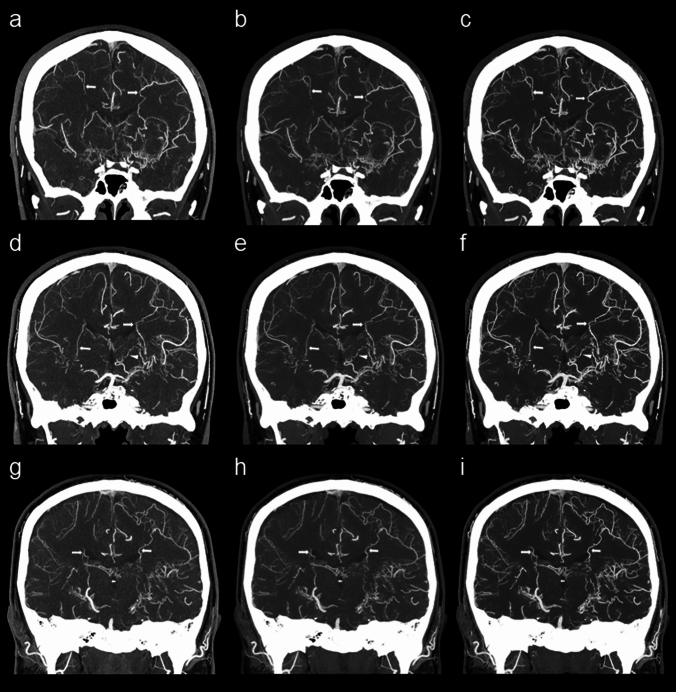
A 42-year-old man with moyamoya disease. Coronal slab MIP images reconstructed using Hybrid-IR (**a, d, g**), DLR-body (**b, e, h**), and DLR-brain (**c, f, i**). The first row (**a–c**) shows bilateral lenticulostriate anastomosis (arrows). The second row (**d–f**) shows left thalamic anastomosis (arrowhead) and bilateral choroidal anastomosis (arrows). The third row (**g–i**) shows bilateral choroidal anastomosis (arrows). *DLR* deep learning reconstruction, *IR* iterative reconstruction, *MIP* maximum intensity projection

Figures 3 and 4 show UHR brain CTA images acquired with each reconstruction algorithm, and Table 2 summarises the results of visual inspection. Interobserver agreement was nearly perfect for each evaluated item (κ = 0.85–0.97). Visual inspection scores of DLR-brain were superior to those of DLR-body and Hybrid-IR for all items (*P* < 0.001). DLR-body was superior to Hybrid-IR for noise magnitude and noise texture (*P* < 0.001), but showed no significant difference in terms of vessel delineation and overall quality.

**Table 2:** Results of qualitative analysis

Visual inspection	Hybrid-IR	DLR-body	DLR-brain
Vessel delineation	4 (4–4)*	4 (4–4)*	5 (5–5)
Noise magnitude	3 (3–3)*^#^	4 (4–4)*	5 (5–5)
Noise texture	3 (3–3)*^#^	4 (4–4)*	5 (5–5)
Overall quality	4 (4–4)*	4 (4–4)*	5 (5–5)

Data are presented as the median (interquartile range)

*DLR* deep learning reconstruction, *IR* iterative reconstruction

^*^Indicates a significant difference from DLR-brain (*P* < 0.001)

^#^Indicates a significant difference from DLR-body (*P* < 0.001)

## Discussion

This study applied DLR-brain to UHR brain CTA in patients with MMD. The performance of this algorithm was assessed and compared with those of Hybrid-IR and DLR-body. In the limited number of previous studies, DLR-brain was reported to reduce image noise and increase CT numbers in brain CTA acquired using conventional multidetector-row CT [22, 23]. To the best of our knowledge, no study has yet assessed the impact of DLR-brain on UHR brain CTA.

In the quantitative analysis, DLR-brain significantly improved edge sharpness, peak CT number, vessel contrast, and FWHM; and reduced image noise (as represented by SD) compared with Hybrid-IR and DLR-body. These findings suggest that DLR-brain provides better contrast between vessels and the surrounding background, as well as clearer vessel edges, thus aiding the identification of vascular location and geometry. In the qualitative analysis, DLR-brain images displayed darker brain parenchyma, providing a favourable background for highlighting intracranial vessels. These vessels appeared brighter and less blurred, which is beneficial for tracing, especially for small or peripheral intracranial vessels. The quantitative and qualitative results were consistent with those of the phantom experiments.

Hybrid-IR and MBIR are known to involve a trade-off between spatial resolution and noise reduction [26, 27]. DLR-brain simultaneously increased spatial resolution and reduced image noise; however, DLR-body did not achieve this. This discrepancy between DLR-brain and DLR-body highlights the importance of using a DLR algorithm optimised for the specific organ being imaged. Given its ability to reduce image noise and enhance spatial resolution compared with DLR-body and Hybrid-IR, DLR-brain has the potential to provide diagnostically sufficient images even at lower radiation doses, which could offer substantial clinical advantages.

The Hybrid-IR algorithm requires selection of a reconstruction kernel. Numerous reconstruction kernels are available for head imaging, including FC41–FC44 without beam hardening correction (BHC), FC20–FC26 (fine grain) with BHC, and FC62–FC68 (coarse grain) with BHC. FC44 demonstrated superior image sharpness in a study that compared the impact of different kernels on the image quality of head and neck CT [28]. It is also the sharpest kernel in the group without BHC and is routinely employed for brain CTA imaging at our institution. Given these characteristics, FC44 was selected for use in the present study. Hybrid-IR reconstructed with FC44 is assumed to provide higher CT numbers and higher spatial resolution than other kernels, making it more challenging to demonstrate a significant difference from DLR-brain.

The majority of previous evaluations of the impact of DLR have used contrast-to-noise ratio to represent vessel contrast [15–19, 21]. Contrast-to-noise ratio requires placement of an appropriate ROI within the vessel boundary, which is difficult to achieve in target vessels of diameter < 1.0 mm. Several studies have reported the profile curve method as a useful tool for quantitative evaluation of small intracranial vessels [9, 23]. Drawing on the experience of these studies, we used the profile curve method in the present quantitative analysis.

Currently, CT is not included in the standard diagnostic imaging for MMD, and the use of CT for diagnosing MMD could be a research topic in its own right. Further evidence is needed to clarify the extent to which DLR-brain may contribute to improved diagnostic accuracy in this context.

This study has several limitations. First, a phantom set at 130 HU was used preferentially for simulation of small blood vessels with equivalent CT numbers. There was a perceptible difference in TTF between results obtained using the phantom set at 350 HU and those obtained at 130 HU. It is notable that even if the former phantom could match the vessel contrast, it could not accurately reproduce details of vessels such as their size and shape. The results obtained in phantoms should be considered to reflect trends rather than providing precise values. As a non-linear reconstruction algorithm, DLR has been reported to change its characteristics in response to changes in contrast [13]. Our TTF results suggest that DLR may have a tendency to improve spatial resolution under higher contrast. Second, all the CT images evaluated were acquired in super-high-resolution mode with a 1024 × 1024 reconstruction matrix. Image noise characteristics have been reported to differ among various acquisition modes of UHR CT and pixel sizes; therefore, the results of this study may not be directly applicable to UHR CT scans using different modes or pixel sizes [29]. Third, the injection protocols varied between paediatric and adult patients. The injection rate was lower for paediatric patients (1 mL/s) than for adult patients (4 mL/s), which could have caused differences in arterial enhancement. Finally, ROI location varied among the patients because the development and diameter of moyamoya perforator vessels varied among the patients, making it infeasible to define a fixed coordinate system or to select vessels based on a constant distance from a specific anatomical landmark. We selected the thinner moyamoya vessels wherever possible. In Fig. 1a, another candidate vessel is located just dorsal to the one selected; however, we chose the thinner of the two for analysis. Therefore, we deduce that almost the same result can be obtained in terms of the superiority of DLR-brain, although the selection of blood vessels affects parameters such as edge sharpness, peak CT number, vessel contrast, and FWHM.

In conclusion, DLR-brain, a new DLR algorithm that is optimised for brain CTA, significantly reduced image noise and improved image quality both quantitatively and qualitatively compared with DLR-body and Hybrid-IR. DLR-brain shows promising potential to enhance the evaluation of vasculature in UHR brain CTA for patients with MMD.
